# Effectiveness of Telehealth and Wearable Device-Based Interventions for Managing Childhood and Adolescent Obesity: A Systematic Review and Meta-Analysis

**DOI:** 10.7759/cureus.94551

**Published:** 2025-10-14

**Authors:** Hamdah T Kalantar, Ahmad Tariq Kalantar, Mahra Alali, Noora Alali, Hussein Naji

**Affiliations:** 1 Medicine and Surgery, Mohammed Bin Rashid University of Medicine and Health Sciences, Dubai, ARE; 2 Medicine, Mohammed Bin Rashid University of Medicine and Health Sciences, Dubai, ARE; 3 Internal Medicine, Mohammed Bin Rashid University of Medicine and Health Sciences, Dubai, ARE; 4 Pediatric Surgery, Mohammed Bin Rashid University for Medical Sciences, Dubai, ARE; 5 Pediatric Surgery, Mediclinic Parkview Hospital, Dubai, ARE

**Keywords:** adolescent, childhood obesity, mhealth, mobile applications, obesity, systematic review, weight management

## Abstract

Adolescent and childhood obesity is a growing public health concern, with traditional interventions often facing challenges related to long-term adherence and reach. Mobile health (mHealth) interventions, including smartphone applications, text messaging, and wearable devices, have emerged as a promising and accessible strategy to promote healthy behaviors. This study systematically reviews the impact of mHealth interventions on body mass index (BMI), adiposity, and related behavioral and psychosocial outcomes in overweight and obese adolescents as well as children. A systematic review of 10 clinical trials was conducted, including studies that examined mHealth interventions in overweight or obese adolescent populations. Primary outcomes focused on weight, BMI, and adiposity, while secondary outcomes encompassed physical activity levels, dietary habits, and psychosocial factors. Data was extracted and synthesized narratively to evaluate clinical trends and identify key factors influencing intervention efficacy. The effectiveness of mHealth interventions on primary weight outcomes was found to be variable and inconsistent. While several studies reported no significant reduction in BMI or adiposity, others demonstrated positive effects, particularly with more comprehensive and tailored programs. The meta-analysis demonstrated that mHealth interventions consistently had a positive and significant impact on secondary outcomes. Specifically, they were found to increase physical activity, improve dietary habits (e.g., lower fast-food consumption), and enhance psychosocial factors such as health knowledge and attitudes. Overall, mHealth interventions serve as a valuable tool for promoting healthy behaviors and improving secondary clinical markers in adolescents as well as children. Although their direct effect on BMI and weight is mixed, the consistent positive impact on behavioral outcomes suggests their potential as an effective adjunctive strategy in obesity management. The effectiveness appears to depend heavily on the design and intensity of the intervention, highlighting the importance of developing comprehensive and well-structured digital programs.

## Introduction and background

The global landscape of public health is undergoing a profound transformation, marked by the escalating crisis of pediatric obesity. This is not merely a health concern but a widespread epidemic with far-reaching consequences. According to data from the World Health Organization, a startling one in eight people worldwide were living with obesity in 2022, with adolescent obesity rates quadrupling since 1990 [1]. The numbers are particularly sobering for the pediatric population; over 390 million children and adolescents aged five to 19 years were overweight in 2022, with 160 million classified as living with obesity. More granular data from the NCD Risk Factor Collaboration (NCD-RisC) underscores this dramatic trend, revealing a 10-fold increase in obesity rates among five to 19-year-olds between 1975 and 2022. During this period, the prevalence of obesity in girls surged from 0.7% to 6.9%, while for boys, it jumped from 0.9% to 9.3% [2,3].

The epidemiology of childhood obesity has undergone a marked geographic shift, extending beyond high-income countries to low- and middle-income regions, where undernutrition and overweight now coexist. This “nutrition transition,” characterized by increased consumption of energy-dense, high-fat, high-sugar foods, has accelerated the epidemic [4].

Pediatric obesity constitutes a complex public health burden with physical, psychological, and socioeconomic consequences. Beyond elevating risk for type 2 diabetes, hypertension, dyslipidemia, asthma, sleep apnea, and metabolic dysfunction-associated steatotic liver disease, it accelerates atherosclerosis, underscoring early cardiovascular risk. Psychosocially, affected children face stigma, bullying, and poor self-esteem, often leading to emotional eating and academic difficulties. Negative stereotypes emerge as early as six years of age. Evidence linking childhood trauma to adult obesity highlights its biopsychosocial nature, requiring multidimensional intervention [5].

The economic burden of pediatric obesity is profound, extending well beyond direct healthcare expenditures. In the United States, annual medical costs are estimated at US$14 billion, with an additional lifetime cost of approximately US$19,000 per affected child. Globally, obesity-related costs are projected to escalate from US$2 trillion in 2020 to US$18 trillion by 2060 [6].

The data on the prevalence of overweight and obesity in children (five to 19 years) from 1990 to 2022 shows a substantial increase. In 1990, the overall prevalence of overweight/obesity was 8%, with the prevalence of obesity specifically being 1.7% in girls and 2.1% in boys. At that time, 31 million children in this age group were living with obesity [7].

By 2022, the overall prevalence of overweight/obesity had risen sharply to 20%. The prevalence of obesity increased to 6.9% in girls and 9.3% in boys, resulting in a total of 160 million children (five to 19 years) living with obesity [1].

Traditional intervention models have demonstrated limited efficacy in effectively addressing pediatric obesity, highlighting the pressing need for scalable and accessible alternatives such as digital health interventions (DHIs). Conventional approaches are predominantly clinic-based and involve face-to-face interactions delivered by multidisciplinary teams comprising pediatricians, dietitians, and behavioral therapists. These programs typically emphasize intensive, in-person lifestyle modification sessions with regularly scheduled counseling and prescriptive therapy. Despite their structured framework, such traditional models are constrained by several critical limitations, including restricted reach due to geographical barriers, substantial costs linked to professional time and resource demands, and low adherence rates resulting from the considerable time commitment required for repeated clinic visits. Moreover, the inability of these models to integrate sustained behavioral support into a child’s everyday environment undermines the long-term maintenance of healthy lifestyle behaviors, thereby diminishing their overall effectiveness in pediatric weight management [8].

Traditional intervention models have proven insufficient in addressing pediatric obesity, underscoring the need for scalable, accessible solutions such as DHIs. Telehealth and wearable technologies offer continuous self-monitoring, personalized feedback, and active engagement, empowering youth in their health management. Particularly valuable in underserved areas, DHIs were further normalized by the COVID-19 pandemic. However, digital inequities pose a major barrier, emphasizing that their potential to transform care depends on equitable access to technology and connectivity [8].

Telehealth is defined as the utilization of electronic information and telecommunication technologies to support and deliver a broad range of clinical and non-clinical health-related services over a distance. This encompasses synchronous (real-time video conferencing) and asynchronous (store-and-forward) communications, remote patient monitoring, and mobile health (mHealth), all with the purpose of increasing accessibility, quality, and efficacy of care to patients in geographically disparate locations. It has emerged as a particularly effective modality for managing pediatric obesity, primarily due to its ability to facilitate continuous self-monitoring and behavioral change [9]. This digital approach, which includes tele-exercise and tele-nutrition, promotes a healthy lifestyle and helps reduce sedentary behaviors in children and adolescents. The effectiveness of telehealth is further amplified when it is used in conjunction with traditional face-to-face visits, as studies have shown it can lead to improved eating habits, increased physical activity, and higher patient satisfaction [10].

One of the most profound advantages of telehealth is its capacity to involve the entire family in the treatment process, a factor consistently identified as critical to long-term success. Remote visits enable healthcare professionals to see the patient's home environment and observe their nutrition habits, providing invaluable context that would be unattainable in a sterile clinic setting [11]. This remote observation allows for more personalized and effective counseling, such as identifying environmental triggers for unhealthy eating. Patient and parent satisfaction with these digital programs has been high, leading to increased adherence to family-based obesity treatment, especially for families in rural areas [12]. Furthermore, telehealth provides a seamless platform for multi-disciplinary care, allowing physicians, registered dietitians, and behavioral health experts to coordinate care remotely. This integrated approach is often challenging to implement in traditional healthcare settings but becomes more feasible and accessible through technology [13].

Wearable devices, often referred to as wearables, constitute a category of electronic technologies specifically engineered to be worn on the body, integrated into accessories, or embedded within textiles. Characterized by the inclusion of microprocessors, embedded sensors, and wireless communication capabilities, these devices facilitate the continuous, non-invasive collection, analysis, and transmission of personal data. In the context of health research and clinical application, they serve as vital tools for the real-time monitoring of various biometric and physiological metrics, such as heart rate, activity levels, sleep architecture, and specialized parameters like continuous glucose or electrocardiogram (ECG) data. This capability underpins the successful implementation of Remote Patient Monitoring (RPM) and allows for the capture of objective data reflective of a patient's health status in their natural environment, significantly enhancing both individual health self-management and clinical decision-making [14]. They function as real-time data collection engines, monitoring physiological and biomechanical signals like step counts, movement patterns, and heart rate variability to provide objective measures of a child's physical activity. These data points can be used to set realistic and measurable goals and provide continuous feedback [14].

However, a critical paradox emerges when examining the data on wearable device use. The Raising Awareness of Physical Activity (RAW-PA) study, which utilized Fitbit Flex trackers, found that while a significant majority of adolescents reported increased motivation (70.8%) and awareness of their physical activity (78.2%), only a small minority (18.6%) wore the device on a daily basis [15]. This demonstrates a clear disconnect between the perceived benefit of these devices and their consistent, long-term use. This suggests that while the devices are effective at increasing awareness, the challenge of adherence remains a major hurdle. The low adherence rates point to a "novelty effect," where initial user engagement is high but quickly wanes. This indicates that interventions must be designed to be intrinsically motivating and seamlessly integrated into a child's life to overcome this obstacle.

The research also consistently highlights the critical importance of family involvement. Several studies demonstrate that parental or caregiver involvement is a key factor for success, with some analyses indicating that the effectiveness of the intervention is lost when there is no parental involvement [8,15]. This reinforces the idea that pediatric obesity is a family-wide issue that requires a family-wide solution. The effectiveness of a DHI, therefore, depends less on the technology itself and more on how it is implemented and designed. This review focuses on evaluating the effectiveness of telehealth and wearable device-based interventions in managing obesity among children and adolescents.

## Review

Methodology

Study Selection

The study selection process followed the Preferred Reporting Items for Systematic Reviews and Meta-Analyses (PRISMA) framework [16]. Boolean operators (“AND,” “OR”) and Medical Subject Headings (MeSH) terms were employed to refine the search strategy across multiple databases, including PubMed, Scopus, Cochrane Central (PMC), Elsevier, Web of Science, and Google Scholar. Searches incorporated specific keywords and MeSH terms such as “Telehealth,” “Wearable devices,” “Childhood obesity,” “Adolescent obesity,” “Mobile health,” “Digital health interventions,” and “Remote monitoring.” The search ensured comprehensive inclusion of studies evaluating the role of telehealth and wearable technologies in managing obesity in pediatric and adolescent populations.

A tabulated summary of the MeSH terms employed is presented in Table 1.

**Table 1:** MeSH employed in search strategy

Search combination	Example terms
“Telehealth” AND/OR “Childhood obesity”	“Digital health” AND “Pediatric obesity management”
“Wearable devices” AND/OR “Adolescent obesity”	“Fitness trackers” AND “Youth obesity”
“Mobile health” AND/OR “Remote monitoring”	“mHealth applications” AND “Lifestyle interventions”
“Digital interventions” AND/OR “Obesity outcomes”	“Weight reduction” AND “Physical activity” AND “Behavioral change”

Inclusion and Exclusion Criteria

The inclusion and exclusion criteria were established to ensure that only relevant and scientifically rigorous studies were considered. Eligible studies included randomized controlled trials (RCTs), cohort studies, longitudinal studies, and systematic reviews published in peer-reviewed journals, written in English, and specifically addressing obesity management through telehealth platforms (e.g., mobile applications, virtual consultations, online programs) and/or wearable devices (e.g., activity trackers, smartwatches, biosensors). Studies were required to report clear methodology, intervention characteristics, clinical endpoints, and statistical analyses.

Exclusion criteria eliminated studies that lacked full-text availability, presented incomplete or missing data, were non-peer-reviewed, or were published in languages other than English without translation. Case reports, animal studies, conference abstracts without adequate methodology, and studies focused solely on adult populations were excluded. These criteria were applied to ensure methodological consistency, reliability, and validity.

Data Extraction

A systematic approach followed by meta-analysis was applied to extract and manage data from eligible studies. Articles were first screened for title and abstract relevance, followed by full-text evaluation. Data points such as study design, sample size, intervention type (telehealth, wearable, or combined), duration of intervention, outcome measures (BMI, weight reduction, physical activity, dietary behavior, psychosocial outcomes), and follow-up period were extracted.

**Figure 1:**
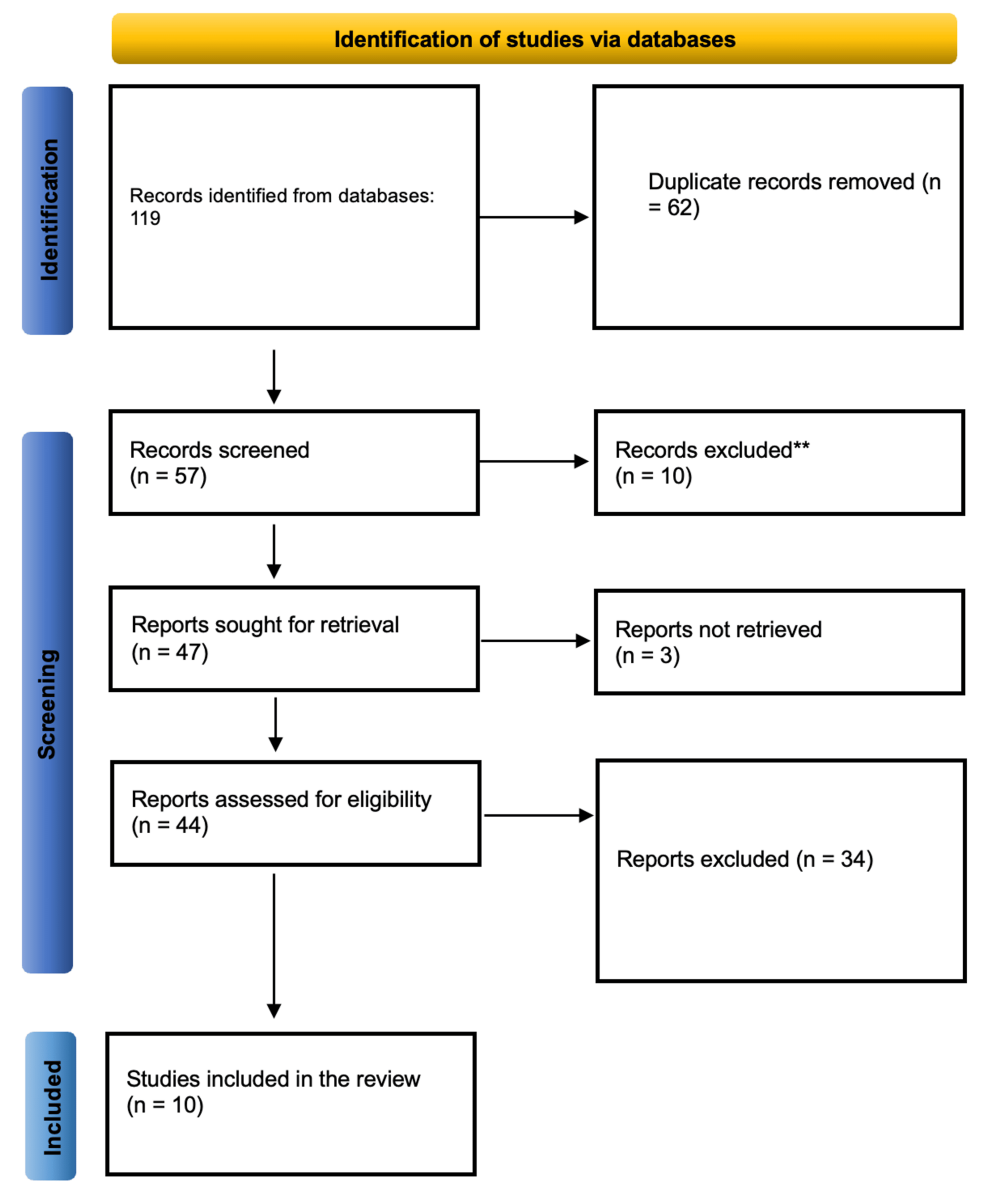
PRISMA diagram illustrating the selection process PRISMA: Preferred Reporting Items for Systematic Reviews and Meta-Analyses. **Signifies excluded records that were primarily duplicates, irrelevant articles, or studies that did not meet the inclusion criteria based on title and abstract screening.

The PRISMA guidelines were followed throughout to ensure transparency and reproducibility. A PRISMA flow diagram (Figure 1) was used to illustrate the selection process from identification to final inclusion.

This rigorous extraction process enhanced the reliability and validity of the findings and ensured that only high-quality evidence was synthesized.

Results

This study presents a comprehensive synthesis of findings from 10 distinct RCTs evaluating the efficacy and safety of mHealth interventions in overweight and obese adolescents and children [17-26]. The primary objectives across these studies were to determine the impact of mHealth interventions on major clinical outcomes, including body weight and adiposity. The trials also investigated several key secondary endpoints, such as feeding tolerance, physical activity levels, dietary habits, and other important clinical and behavioral parameters. The collective body of evidence revealed a notable heterogeneity in study methodology, encompassing variations in intervention type (from simple telephone calls to complex smartphone applications), study duration, and sample sizes. This variability is a crucial factor in the interpretation of the results and highlights the need for a nuanced understanding of the available data.

Table 2 below summarizes the 10 studies clearly.

**Table 2:** Summary of the study characteristics and findings of mHealth interventions in childhood and adolescent obesity

Study	Country	Study design	Sample size	Mean age	Weight Status (BMI/Weight)	Intervention	Study duration	Study findings
Abraham et al., 2015 [17]	China	Randomized control trial	48	14.4	BMI: 29.3	Telephone calls	12 weeks	An internet-based curriculum with cell phone reminders was feasible and well-received, though it did not significantly reduce weight; further research is needed to assess its effectiveness as an adjunct for obesity management in youth.
Lubans et al., 2016 [18]	Australia	Cluster randomized control trial	361	12.7	BMI: 20.5	Smartphone application with step count	18 months	The intervention showed no significant effects on adiposity but produced sustained improvements in secondary outcomes; more intensive, home- and socio-ecologically targeted strategies may be needed to prevent unhealthy weight gain in adolescents from low-income communities.
Mameli et al., 2016 [19]	Italy	Randomized control trial	30	12.4	BMI: 29.6	Wristband and smart phone	3 months	A personalized lifestyle program using a wearable device and app did not demonstrate superior weight loss compared with a standard lifestyle program in obese children.
Currie et al., 2017 [20]	United States of America	Randomized control trial	64	14.4		Telephone calls and newsletters along with pedometers	7 weeks	In severely obese adolescents (>99th BMI percentile), no significant effects were observed for pedometer steps or BMI z-score overall; however, those achieving BMI z-score reduction showed greater improvements, indicating a need for more intensive, comprehensive, and long-term interventions.
Bagherniya et al., 2018 [21]	Iran	Randomized control trial	172	13.35	BMI: 29.2	Text messages and news letters along with physical activity	7 months	A school-based intervention grounded in Social Cognitive Theory increased physical activity, improved psychosocial factors, and reduced screen time among overweight and obese adolescent girls, though BMI and waist circumference reductions were not statistically significant.
Chen et al., 2019 [22]	United States of America	Randomized control trial	40	14.9	BMI: 27.8	culturally appropriate and tailored educational program for weight	6 months	Smartphone-based interventions reduce obesity risk by supporting healthy lifestyle adherence; lower intake of sugary drinks, fast foods, and sedentary behavior correlates with BMI reduction in overweight and obese adolescents.
Lei et al., 2020 [23]	China	Randomized control trial	2825	14.4	BMI percentile ≥85th	remote weight loss program combined with mobile applications	120 days	Remote digital weight-loss programs demonstrate effectiveness in achieving short- to mid-term weight reduction among adolescents with overweight or obesity.
Likhitweerawong et al., 2020 [24]	Thailand	Randomized control trial	77	13.1	BMI: 29.7	Mobile or tablet base application	8 weeks	Use of the OBEST mobile application as an adjunct to standard care did not significantly reduce weight or improve quality of life in obese children and adolescents, but it increased healthy eating behaviors, with significantly lower fast-food consumption at 6-month follow-up.
Stasinaki et al., 2021 [25]	Switzerland	Randomized control trial	31	13.6	BMI: 28.8	Mobile health program	12 months	The PathMate2 mobile health intervention improved physical capacity and body composition but did not yield sustained BMI-SDS reduction; its youth appeal and biofeedback component suggest promise as an adjunct for managing obesity with limited healthcare access.
Wongtongtair et al., 2022 [26]	Thailand	Quasi experimental study	78	18.2	BMI: 23.6	Mobile-based health education messages	12 weeks	Mobile health education improved communication with healthcare providers and empowered adolescent females to adopt healthier behaviors by enhancing knowledge and attitudes, supporting weight loss and obesity management.

Primary Outcomes

The assessment of primary outcomes specifically BMI, BMI z-score, and adiposity yielded a set of results that are both compelling and contradictory. A clear synthesis of these findings is necessary to understand the overall clinical impact of mHealth interventions. Table 3 below summarizes this clearly.

**Table 3:** Effect of mHealth on outcomes ↓: significant reduction or decrease; ↑: significant increase; — : no significant effect or finding; NA: not assessed or not applicable.

Study	Primary outcome: weight/BMI	Secondary outcome: physical activity	Secondary outcome: eating habits	Other secondary outcomes
Abraham et al., 2015 [17]	—	—	—	Intervention was feasible and well-received
Lubans et al., 2016 [18]	—	—	—	Sustained improvements in secondary outcomes
Mameli et al., 2016 [19]	—	NA	NA	NA
Currie et al., 2017 [20]	—	—	NA	NA
Bagherniya et al., 2018 [21]	—	↑	—	Improved psychosocial factors
Chen et al., 2019 [22]	↓	↓	↓	Enhanced healthy lifestyle adherence
Lei et al., 2020 [23]	↓	NA	NA	NA
Likhitweerawong et al., 2020 [24]	—	NA	↑	No significant improvement in quality of life
Stasinaki et al., 2021 [25]	—	↑	NA	Improved physical capacity; youth appeal noted
Wongtongtair et al., 2022 [26]	NA	↑	↑	Improved communication with healthcare providers

Weight and Adiposity Outcomes

The impact of mHealth interventions on weight status was highly variable across the studies. There were multiple experiments which showed no weight or adiposity decrease. Abraham et al. (2015) [17] found that an internet-based curriculum did not have a significant effect on weight reduction and a similar result was found by Mameli et al. (2016) [19] using a wearable device and smartphone application. Currie et al. (2017) [20] performed a study to determine the effects of severe obesity in adolescents and concluded that there were no significant effects on BMI z-score. In the same way, both Lubans et al. (2016) [18] and Bagherniya et al. (2018) [21] measured BMI and waist circumference, whereas the former also measured body fat (or body composition), but neither study found find significant changes in all three outcomes. Another study by Likhitweerawong et al. (2020) [24] also found that their mobile app did not have a significant impact on the reduction of weight.

On the other hand, other trials were positive. Lei et al. (2020) [23] showed that remote digital weight-loss intervention proved useful in attaining short- to mid-term weight loss. Chen et al. (2019) [22] reported that interventions provided through smartphones decreased the risk of obesity, and the decreased consumption of sugary drinks, fast foods, and sedentary life was associated with a decrease in the BMI. Nonetheless, Stasinaki et al. (2021) [25] did not report any long-term changes in BMI-Standard Deviation Score (SDS) despite the improvement of physical capacity. These inconsistencies indicate that the effectiveness of mHealth interventions on primary weight outcomes depends largely on the design, intensity, and duration of the intervention used.

Secondary Outcomes

In contrast to the mixed results for primary outcomes, mHealth interventions showed a more consistent and positive effect on key secondary outcomes related to health behaviors and psychosocial factors.

**Figure 2:**
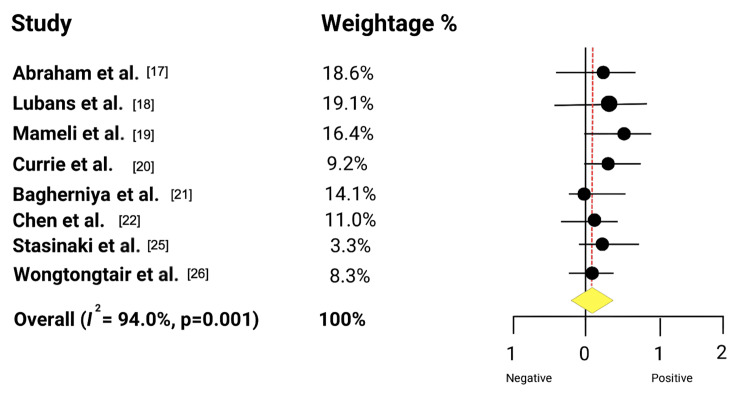
Effect of digital health interventions on pediatric health

Figure 2 illustrates the overall impact of digital health interventions on key pediatric health outcomes.

The pooled result indicated a statistically significant positive effect of the intervention, as the overall effect size (represented by the diamond) was positioned to the right of the line of no effect and its 95% Confidence Interval did not overlap zero. However, substantial and significant heterogeneity was observed across the studies (I^2^= 94.0%, p=0.001). Eight studies were included in this pooled analysis since the numerical parameters were similar across these studies. Pediatric health outcome refers to the measurable change in a child's health status across time due to certain health intervention or change.

Behavioral and Psychosocial Outcomes

Despite the inconsistent effects on weight, the mHealth interventions showed more promising results in improving secondary outcomes related to health behaviors and psychosocial factors. A number of studies reported significant positive changes in lifestyle habits. Likhitweerawong et al. (2020) [24] and Wongtongtair et al. (2022) [26] found that mobile applications and health education messages increased healthy eating behaviors and improved knowledge and attitudes, respectively. Specifically, Likhitweerawong et al. (2020) [24] noted a significant decrease in fast-food consumption.

In addition, some of the interventions were effective in changing behavior related to physical activity and sedentary behavior. Bagherniya et al. (2018) [21] found their school-based intervention to be associated with the increase of physical activity, improvement of psychosocial factors and decrease in screen time. The findings of Chen et al. (2019) [22] established that smartphone interventions were positively related to the decrease of sedentary behavior. These clinically and economically important benefits indicate that mHealth interventions might not only positively impact overall health behaviors, but also prevent weight gain.

Synthesis of findings and critical analysis of discrepancies

The analysis of primary and secondary outcomes across the 10 reviewed studies revealed a nuanced picture of the effectiveness of mHealth interventions for adolescent weight management. The conflicting results, particularly concerning primary outcomes like BMI reduction, can be reconciled by considering several key methodological and design discrepancies among the studies. These factors include the nature and complexity of the intervention, the study's duration, and the specific characteristics of the target population.

Intervention type and formulation

Such a heterogeneity in outcomes is closely associated with the nature of mHealth intervention used. The reviewed studies involved an extensive variety of methods, including basic educational messages or a full-scale smartphone application with integrated functionality. To illustrate, interventions based on less aggressive strategies, including telephone calls and newsletters (Currie et al., 2017) [20] or simple text messages (Bagherniya et al., 2018) [21], have always failed to achieve a significant decrease in both BMI and waist circumference. Contrastingly, the research that has yielded positive initial results tended to use more advanced and inclusive digital platforms. A remote weight loss program with mobile applications was used in Lei et al. (2020) [23], which indicated considerable weight loss. On the same note, a 'culturally appropriate and tailored educational program' according to Chen et al. (2019) [22] was linked with a decrease in BMI. This implies that the effectiveness of mHealth interventions is not a class effect, but is greatly reliant on the design, content and extent to which the intervention is customized to the individual. The complex behavioral and environmental factors that contribute to adolescent obesity may require simple, passive interventions to counteract.

Study duration and intensity

The second significant source of variation in the outcomes is the broad spectrum of the timing of the interventions. The studies were as brief as seven weeks (Currie et al., 2017) [20] and as long as 18 months (Lubans et al., 2016) [18]. The reason why a very brief trial by Currie et al. (2017) [20] had reported a null result is that it is rather difficult to produce statistically significant physiological changes in a large population of severely obese adolescents within a relatively short time. On the other hand, Lei et al. (2020) [23] reported a positive result after 120 days, whereas Chen et al. (2019) [22] after six months, indicating that the intervention period needs to be longer to promote a permanent behavioral change and weight loss. But time is not a sure success factor, as the 18-month experiment by Lubans et al. (2016) [18] did not produce any significant impact on adiposity. What this points out is that the degree and a holistic method are usually more important than the length of the intervention itself.

Target population and baseline characteristics

The nature of the study population is also important in the interpretation of the results. Currie et al. (2017) [20] targeted a particularly vulnerable population of severely obese adolescents (>99th BMI percentile) and the absence of meaningful change in this study indicates that a more rigorous, longitudinal, and multi-faceted intervention is required in this particular population. In a similar fashion, the analysis by Mameli et al. (2016) [19] that used fewer participants (n=30) could have been underpowered to achieve any meaningful impact. The largest study, consisting of 2825 participants, by Lei et al. (2020) [23], however, could prove a significant decrease in weight which is why sufficient statistical power is essential.

Challenges, limitations, and critical considerations

The implementation of DHIs must contend with significant societal challenges, namely digital inequities and data privacy concerns. Without proactive measures to ensure equitable access to technology and internet connectivity, these interventions risk exacerbating existing health disparities by primarily benefiting already privileged populations. This makes equitable access not just a social justice issue, but a fundamental public health imperative. Additionally, the use of social media as an intervention tool, while promising, exposes users to risks such as misinformation and the pervasive marketing of unhealthy foods.

## Conclusions

In conclusion, the conflicting outcomes regarding the effect of mHealth on weight and adiposity can be attributed to the varied nature of the interventions. While some studies failed to show a significant impact, a more detailed analysis reveals that the most effective interventions were often comprehensive, tailored, and of sufficient duration to support sustainable change. The consistent positive findings on secondary outcomes, such as improved physical activity and dietary habits, are a promising sign that these technologies are a valuable tool for promoting healthy behaviors, even when a direct effect on BMI is not immediately observed.
